# DFT Study on the Enhancement of Isobaric Specific Heat of GaN and InN Nanosheets for Use as Nanofluids in Solar Energy Plants

**DOI:** 10.3390/ma16030915

**Published:** 2023-01-18

**Authors:** Francisco Moreno-Velarde, Elisa I. Martín, José Hidalgo Toledo, Antonio Sánchez-Coronilla

**Affiliations:** 1 Departamento de Química Física, Facultad de Farmacia, Universidad de Sevilla, E-41012 Sevilla, Spain; 2 Departamento de Ingeniería Química, Facultad de Química, Universidad de Sevilla, E-41012 Sevilla, Spain

**Keywords:** GaN, InN, DFT, ELF, QTAIM, isobaric specific heat

## Abstract

In this work, GaN and InN nanosheets with dodecylamine (DDA) as surfactant have been studied as nanofluids to be used in solar plants. The interactions between the sheets and the surfactants have been performed using density functional theory. The most favorable interaction site on the surface corresponds to the metallic atom of the sheet with the N atom of the surfactant. In this interaction, the pair of electrons of N from the surfactant with the metal atom of the sheet play a stabilizing role, which is corroborated by electron localization function (ELF), quantum theory of atoms in molecules (QTAIM), and density of states (DOS) analysis. The isobaric specific heat values for the most favorable interaction were obtained in the presence of water, ethylene glycol, and diphenyl oxide as solvents for the first time. The highest value corresponds to systems with diphenyl oxide, being the values obtained of 0.644 J/gK and 0.363 J/gK for GaN-DDA and InN-DDA systems, respectively. These results open the possibilities of using GaN-DDA and InN-DDA systems in solar energy applications.

## 1. Introduction

Energy is one of the main factors involved in economic development and is relevant in increasing the wealth of a country. One of the many concerns of modern society is the increasing demand for electricity on the planet. As the population increases, so does electricity consumption, and it is necessary to keep up with demand in order to avoid power shortages and overall increase the quality of life of the population [1,2].

Most traditional processes used to generate electricity often involve burning large amounts of fossil fuels, which in turn negatively affect the environment. As the environmental situation worsens, the supply of fossil fuels becomes scarcer, and the energy demand grows yearly, the scientific community is approaching greener solutions, the so-called alternative or renewable energy sources, which reduce greenhouse emissions and minimize environmental harm. Renewable sources of energy are those coming from natural occurring phenomena such as the kinetic energy coming from the movement of the wind or water, or in this case, the thermal energy from the Sun. Human beings have been able to harness these different renewable sources to transform them into usable energy, thus replacing coal and other fossil fuels. Solar energy stands out as a very interesting source of energy due to its availability (irradiance of the sun is around 1 kW/m^2^ in the surface of Earth) and high exergy (amount of energy available for potential use before reaching the equilibrium) [1,2,3]. Concentrating solar power (CSP) plants it is clean energy with a great potential to supply the global energy demand [4]. However, this renewable energy has still a high cost compared to fossil-based electricity, which is its main drawback. Therefore, increasing the efficiency of these CSPs may solve that economic inconvenience. The efficiency of a solar collector can be enhanced by using thermal and conductive fluids, generally thermal oils. These thermal oils act as heat transfer fluids (HTFs), transforming radiation into thermal energy to be used to produce electricity [5,6,7].

The use of nanofluids (solid nanoparticles in a fluid) can improve the thermal properties of the base fluid as specific heat, conductivity, and heat transfer coefficient [5,8,9]. The use of nanofluids with metal nanoparticles (Cu, Ag, Au) leads to good heat transfer performance [10,11,12,13] up to 20% increase for Pt [14], as well as the use of metal oxides such as TiO_2_, NiO and Al_2_O [5,7,13,15,16]. Theoretical analysis of the thermal properties of metal nanoparticles and metal oxides in the presence of Dowtherm A as HTF and different surfactants reveals the relevant role that the surfactant and diphenyl oxide structure from HTF plays in the thermal properties of the first layer of molecules around the center of mass of the nanoparticle [5,6,10,11,12,14,15]. Recent studies with MoS_2_, WS_2_, WSe_2_ and graphene-type materials with two-dimensional structure show an increase in thermal properties due to the large surface area of the sheets [17,18,19,20,21,22]. Furthermore, the effect of surfactant in chalcogenide systems is very important because it can be obtained in nanowires or nanosheets [21]. So, the effect of surfactant plays a key role in the stability of sheets, as well as a notable role from the molecular level in thermal properties. Nitrides from the 13 Group, such as BN, have interesting properties for being obtained as nanowires, nanosheets, and used as nanofluids with good thermal performance [23,24]. Recent studies on GaN and InN sheets have shown their feasibility in being used as sensors by adsorption of molecules on their surfaces [25,26], as well as for solar photovoltaic energy conversion [27], which opens scientific interest in exploring its properties if used as nanofluids. In this work, GaN and InN sheets were selected, and their interactions with dodecylamine (DDA) as a surfactant were studied for the first time. The study of the interactions with the surfactant aims to explore the stability of the nanofluid. Thus, the most favorable interaction site has been explored, which presents a deep structural and electronic analysis to understand the stability. The study of isobaric specific heat is focused on testing the viability of these systems for their use in solar plants. Therefore, the isobaric specific heat for GaN and InN with DDA in the presence of water, ethylene glycol, and diphenyl oxide has been analysed, making the systems suitable for use as nanofluids in solar plants.

## 2. Computational Framework

Density functional theory (DFT) calculations have been carried out. The B3LYP functional with Landl2dz pseudo potential for the optimization of the structures and DGDZVP basis set for the atom in molecules analysis were used as implemented in Gaussian 09 [28]. The inclusion of solvent has been used with the integral equation formalism (IEF) of the polarisable continuum model (PCM) with water, ethylene glycol, and diphenyl oxide as solvents [28,29,30]. AIMAII software package [31] was used for the wavefunction analysis within the AIM theory. This quantum chemistry software package is a package for performing comprehensive QTAIM analyses of molecular systems [31]. Viena ab Initio software package was used for periodic calculation with electron exchange and correlation treated within the generalized gradient approximation with PBE as functional [32,33,34,35]. The number of plane waves in this software was controlled by a cutoff energy; in the present study, a cutoff of 600 eV was applied [32,33,34,35]. The GaN sheet was prepared using Nanotube modeler, and its structure was used as representative for the InN sheet [36]. For the electron localization function (ELF) analysis [37,38,39,40] analysis Vaspview software was used [41]. Chemcraft was used for the structure images [42].

## 3. Results and Discussion

**Figure 1:**
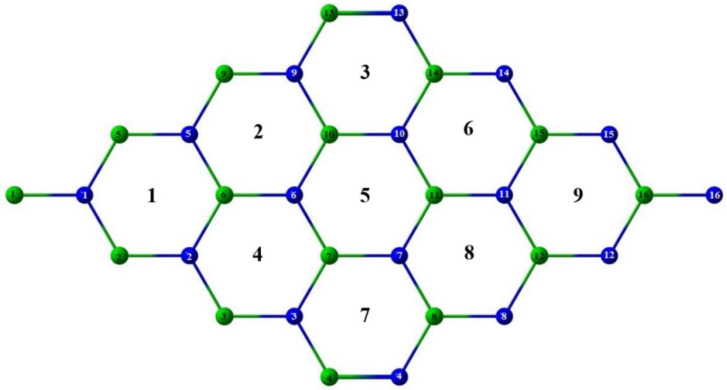
Sheet structure. Green: Ga/In. Blue: N.

The interactions between DDA as surfactant with GaN and InN sheets have been studied. The selected sheet has 32 atoms composed of 16 atoms of Ga or In with 16 N atoms, that is, Ga_16_N_16_ and In_16_N_16_. The structure of the sheet is shown in Figure 1, where the green and blue spheres represent the Ga/In and N atoms, respectively. The sheet consists of nine rings numbered from 1 to 9. Both the metallic and nitrogen atoms are numbered from 1 to 16 as shown in Figure 1. 

**Figure 2:**
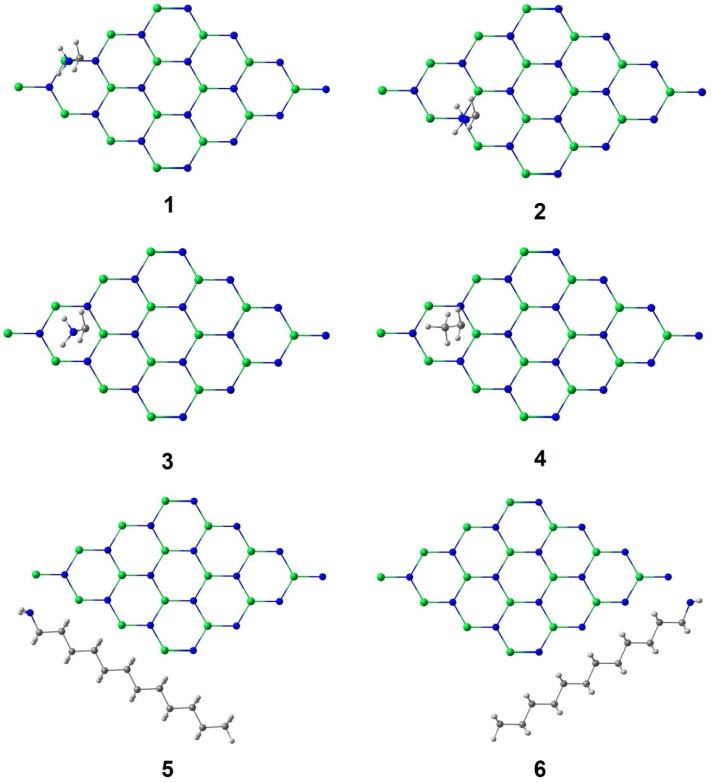
Structures selected for interactions between the surfactant and the sheet. Green: Ga/In. Blue: N. White: H. Grey: C.

Six representative interaction sites between the surfactant and the sheet have been studied. These interaction sites are shown in Figure 2. Thus, the energetic results will help reveal the most favorable interaction site and how the surfactant is placed in relation to the sheet. For clarity purposes, only the terminal atoms of the DDA are shown in positions **1**–**4**. Positions **1**, **2**, and **3** imply the interaction of the N atom from DDA with Xx (Xx=Ga, In), N, and placed over the centre of the ring, respectively. Position **4** places the methyl terminal of the DDA chain over the centre of the ring. Sites **5** and **6** correspond to the interaction of the surfactant chain along the sheet with the N atom that is focused on atom 1 Xx or N 16, respectively.

The interaction energies (*E_int_*) associated with the six binding sites in Figure 2 are obtained as the difference between the energy of the sheet with the surfactant ($E_{\left(sheet+surfactant\right)}$) with the energies of the sheet ($E_{\left(sheet\right)}$) and surfactant ($E_{\left(surfactant\right)}$) as it is defined in Equation (1)

(1) $$E_{int}=E_{\left(sheet+surfactant\right)}-E_{\left(sheet\right)}-E_{\left(surfactant\right)}$$

**Figure 3:**
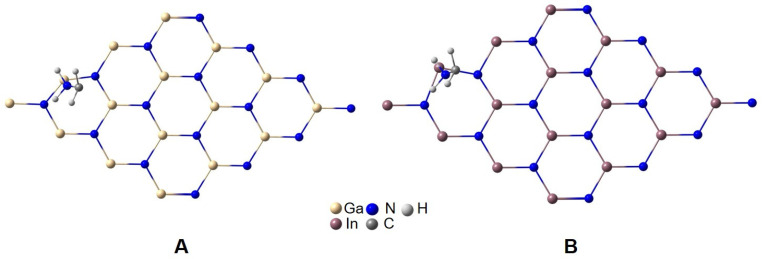
Optimized structures for position **1** for GaN-DDA (**A**) and InN-DDA (**B**) systems.

Table 1 shows the interaction energy values for both the GaN-DDA and InN-DDA systems, respectively. The most favorable interaction site corresponds to position **1**, where the N atom of the surfactant is placed over the Ga and In atoms, followed by that of the surfactant placed on top of N (**2**). Binding sites **3**, **4**, **5** and **6** are less stable, but the hydrogen bond between H atoms from DDA and N atoms from the sheet may explain the lowest value for the interactions in position **6**. Based on energy considerations, the structures corresponding to position **1** for both the GaN-DDA and InN-DDA systems will be selected for discussion from now on (Figure 3). 

### 3.1. GaN-DDA and InN-DDA Systems

#### Geometrical and Electronic Analysis

**Figure 4:**
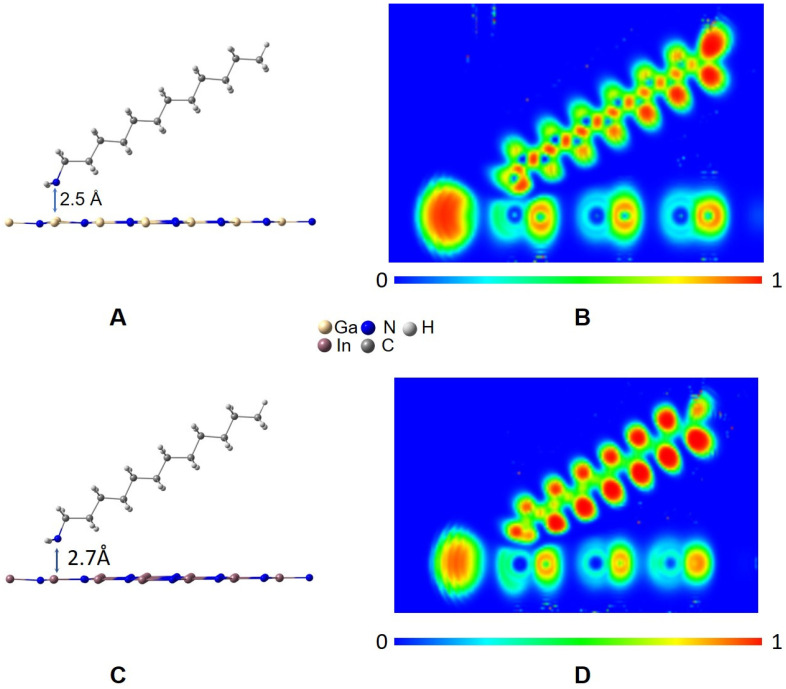
Optimized structures and ELF analysis for GaN-DDA (**A**,**B**) and InN-DDA (**C**,**D**) systems.

**Figure 5:**
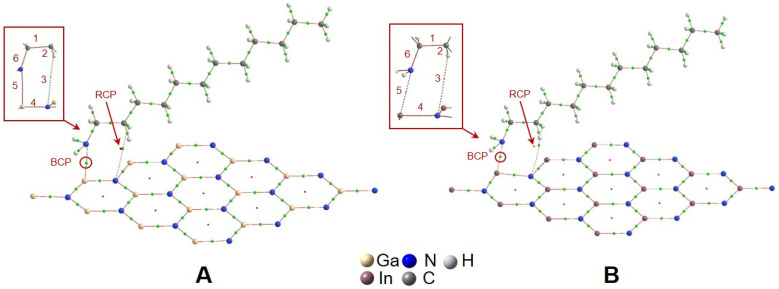
QTAIM analysis for GaN-DDA (**A**) and InN-DDA (**B**) systems.

Figure 3 and Figure 4A,C show the optimized structure for position 1 in the GaN-DDA and InN-DDA systems. The interaction of the N atom from DDA with Ga from the GaN sheet implies an attraction between both atoms with the Ga atom 8º out of the sheet plane and a Ga-N distance around 2.5 Å (Figure 4A). This interaction is also observed in the InN system with the In atom 5º out of the sheet plane and an In-N distance of 2.7 Å (Figure 4C). In this attraction, the lone electron pair from the N atom of DDA plays a crucial role, as shown in the ELF analysis (Figure 4B,D). In Figure 4B,D, the ELF image is focused on the N atom from DDA. In Figure 4B,D, it can be seen how the electron cloud (orange-red colour) is directed toward both the Ga and In atoms of the sheet. In the case of the GaN-DDA system (Figure 4B), the green and blue halos from N of DDA and Ga of the sheet are nearly in contact, respectively, which means that this interaction is more favoured than the same for the InN-DDA system. An interesting explanation of this interaction can be found by applying the quantum theory of atoms in molecules (QTAIM) to the optimized geometry [43,44]. According to this theory, which is based on the topological analysis of the density, a bond critical point (BCP) is found along the bond path between the Ga and In atoms of the sheets and the N atom from the DDA. This BCP is shown in both systems as a green point with a red circle in the molecular graphs of Figure 5. At this BCP, the values of electron density (*ρ*) and its Laplacian (∇^2^*ρ*) are 0.0313 a.u. and 0.1075 a.u. for GaN-DDA (Figure 5A). Those low and positive values for density and Laplacian are typical of closed-shell interactions such as van der Waals or ionic that stabilize the system [43,44]. For InN-DDA (Figure 5B), the values of *ρ* (0.0218 a.u.) and ∇^2^*ρ* (0.0752 a.u.) are a bit lower than those of GaN-DDA, indicating that the GaN-DDA interaction is slightly more favoured. Furthermore, according to theory, stabilizing ring critical points (RCPs) are found in the interior of the bond paths that form a ring (red point in the QTAIM analysis of Figure 5) [44]. It is worth mentioning that a RCP is found inside the six-membered ring formed between the bond paths of the surfactant and the GaN/InN sheet (Figure 5). This six-membered ring is shown in the red squares of Figure 5. Thus, the presence of this RCP also stabilizes the GaN-DDA and InN-DDA systems.

**Figure 6:**
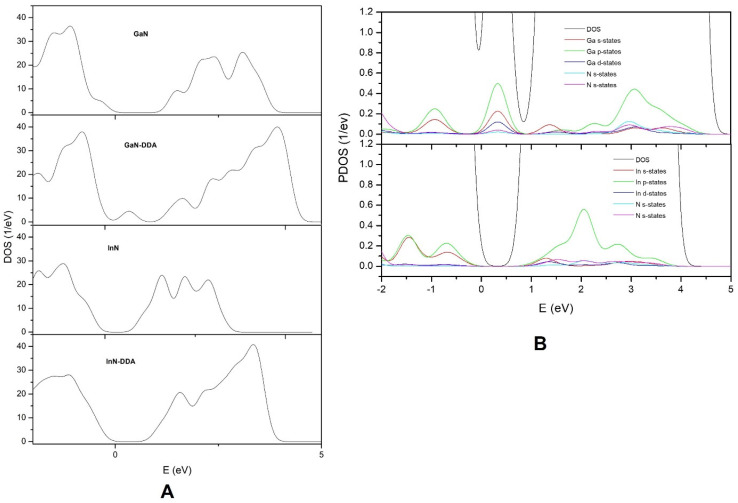
DOS of the structure for GaN and InN with and without DDA at position **1** (**A**). PDOS for the projected states of N, Ga and In atoms at position **1** for GaN-DDA and InN-DDA, respectively (**B**). The Fermi level is set to 0.

Finally, the electronic interaction between the metallic and N atoms of the sheet and the DDA, respectively, is qualitatively described by partial density of states (PDOS) analysis (Figure 6B). Figure 6A shows the density of states of the GaN sheet and the GaN-DDA system. The presence of the surfactant can be observed to imply the appearance of new bands above the Fermi level. Figure 6B shows the PDOS over the s and p states of both.

The N and Ga atoms and Ga d states are also included. The overlapping observed between the states of N and Ga corroborates the interaction between both atoms, in agreement with the previous discussion from the ELF and QTAIM analysis. A similar scenario is observed for the DOS and PDOS of the InN-DDA system. The estimated band gap for the InN system is around 0.6 eV, which is lower than that for GaN (c.a. 1.1 eV). The presence of surfactant modifies the structure of the conduction band compared to that of the InN sheet (Figure 6A). The PDOS analysis of the InN-DDA system also reveals an overlap between the states s and p of the N and In atoms (Figure 6B). This result is indicative of the interaction between the N and In atoms, which agrees with the description of ELF and QTAIM.

### 3.2. Isobaric Specific Heat for GaN-DDA and InN-DDA Systems

**Figure 7:**
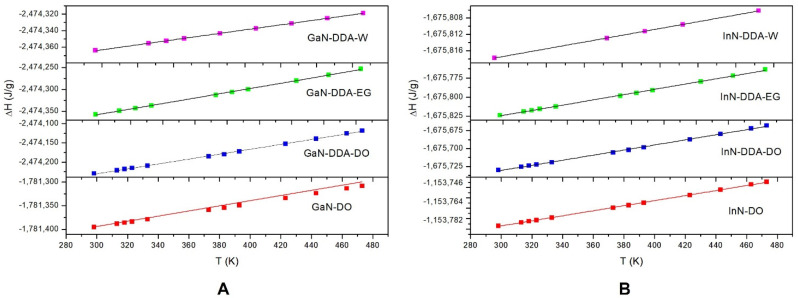
Plot of the enthalpy versus temperature for the GaN (**A**) and InN (**B**) systems in the presence of water (W), ethylene glycol (EG), and diphenyl oxide (DO) as solvents.

The isobaric specific heat (C_p_) is a thermal property of relevance for the election of a nanofluid, so, high values of C_p_ are preferable in CSPs. The isobaric specific heat for the GaN-DDA system in the presence of water (W), ethylene glycol (EG), and diphenyl oxide (DO) as heat transfer fluids (HTF) has been studied. Diphenyl oxide has been selected as representative of the base fluid because it has the highest proportion in the eutectic mixture of commercial Dowtherm A fluid, that is, a mixture of biphenyl (26.5%) and diphenyl oxide (73.5%). This fluid from Dow Chemical Company is selected because it is commonly used as HTF in central plants. The C_P_ is calculated as the slope of enthalpy versus temperature. This methodology has been validated in previous works [11,12,14,15]. Figure 6 shows the evolution of energy versus temperature in the presence of W, EG, and DO. To qualitatively validate the effect of the inclusion of surfactant in the system, we compared the C_P_ values obtained for GaN and InN sheets (0.497, 0.243 J/gK, respectively) and the GaN-DDA and InN-DDA systems (0.644, 0.363 J/gK, respectively) with diphenyl oxide as solvent (Figure 7). Therefore, the presence of DDA clearly enhances the C_P_ values of the system, with the values of the system with Ga being higher than those with In. In fact, a general trend is observed for both systems in the presence of W, EG, and DO as solvents. For the GaN-DDA system in the presence of solvents, the C_P_ tendency is as follows: DO (0.644 J/gK) > EG (0.628 J/gK) > W (0.604 J/gK). For InN-DDA, the tendency is DO (0.363 J/gK) > EG (0.354 J/gK) > W (0.332 J/gK). Thus, the highest values correspond to the systems in the presence of DO, followed by EG and W. Although the C_P_ values are similar, the highest value is obtained when diphenyl oxide is used as the solvent. In fact, the GaN-DDA-DO system with the highest C_p_ may be proposed for use in solar energy applications. 

## 4. Conclusions

A theoretical study of the interactions between DDA as surfactant with GaN and InN sheets has been performed. Different interaction sites have been analysed. For both systems, the most favorable interaction site corresponds to that of the terminal N of the surfactant placed over the Ga and In atoms of the sheet. The interaction implies a reorganization of the structure around the studied site with an attraction detected between the metallic atoms of the sheet and the N atom of the surfactant, represented by 8 and 5 degrees out of the sheet plane for the Ga and In atoms, respectively. In this attraction, the pair of electrons of N from the surfactant plays a relevant role, as it is corroborated by the DOS and ELF analysis. Moreover, the QTAIM analysis reveals the electronic stabilization of the systems with surfactants with both typical close-shell interactions and the presence of ring critical points that favor the stabilization of those interactions. Finally, the study of isobaric specific heat in the presence of solvents shows that the highest value corresponds to the systems with DO, as well as the Cp values of GaN-DDA (0.644 J/gK) > EG (0.628 J/gK) > W (0.604 J/gK) higher than those of InN-DDA (0.363 J/gK) > EG (0.354 J/gK) > W (0.332 J/gK). Thus, the systems studied can be proposed to be used in solar energy applications.

As a summary, the main points of the current research are shown as abbreviated sentences in the following:

- GaN and InN sheets interact with DDA as surfactant, preferably with the terminal N of the surfactant placed over the Ga and In atoms of the sheet.
- The highest value of isobaric specific heat corresponds to the systems with DO used as representative of a commercial HTF.
- GaN and InN sheets can be proposed to be used in CSPs.

## Figures and Tables

**Table 1:** The energy of interaction associated with each binding site is shown in Figure 2.

	*E_int_*/eV	
Position	GaN-DDA	InN-DDA
**1**	−0.704	−0.655
**2**	−0.143	−0.134
**3**	0.524	1.403
**4**	0.866	1.142
**5**	1.031	1.140
**6**	0.203	0.225
